# Early and repeated screening detects children with persistent attention-deficit/hyperactivity disorder

**DOI:** 10.1007/s00787-023-02284-8

**Published:** 2023-08-25

**Authors:** Kristin Romvig Overgaard, Beate Oerbeck, Svein Friis, Are Hugo Pripp, Heidi Aase, Guido Biele, Christine Baalsrud Ingeborgrud, Guilherme V. Polanczyk, Pål Zeiner

**Affiliations:** 1 Division of Mental Health and Addiction, Oslo University Hospital, Nydalen, PO box 4959, 0424 Oslo, Norway; 2 Institute of Clinical Medicine, University of Oslo, Oslo, Norway; 3 Oslo Centre of Biostatistics and Epidemiology, Oslo University Hospital, Oslo, Norway; 4 Department of Child Health and Development, Norwegian Institute of Public Health, Oslo, Norway; 5 Faculdade de Medicina FMUSP, Department of Psychiatry, University of Sao Paulo, Sao Paulo, Brazil

**Keywords:** Persistent, ADHD, Preschool, Children, Longitudinal

## Abstract

Preschool screening of attention-deficit/hyperactivity disorder (ADHD) has been found too inaccurate to be clinically useful. This may be due to the known instability of ADHD symptoms from preschool onwards, and the use of a single screening only. We hypothesized that by identifying a group of children with persistent ADHD from preschool to school age and repeating the screening, the clinical usefulness of screening would increase. This study is part of the prospective longitudinal, population-based Norwegian Mother, Father and Child Cohort Study, with a diagnostic parent interview at 3.5 years and follow-up with parent questionnaires at ages 5 and 8 years (*n* = 707). We identified a group classified with ADHD at all three time points (persistent ADHD). We then used the Child Behavior Checklist ADHD DSM-oriented scale at ages 3.5 and 5 years to investigate the accuracies of single- and two-stage screening at different thresholds to identify children with persistent ADHD. About 30% of the children were classified with ADHD at least once across time (at ages 3.5, 5, and/or 8 years), but only 4% (*n* = 30) had persistent ADHD. At all thresholds, the two-stage screening identified children with persistent ADHD more accurately than single screening, mainly due to a substantial reduction in false positives. Only a small group of children were classified with persistent ADHD from preschool to school age, underlining that future screening studies should distinguish this group from those with fluctuating symptoms when estimating screening accuracies. We recommend a two-stage screening process to reduce false positives.

Attention-deficit/hyperactivity disorder (ADHD) with persisting symptoms of hyperactivity–impulsivity and inattention often debuts in early childhood, yet is usually diagnosed after school entry [1, 2]. This is unfortunate, because early identification has been underlined as critical to the well-being of children and their families [3] and may alleviate the socioeconomic impact and burden of ADHD on society [4]. However, a recent meta-analysis concluded that future work is required to develop the most efficient strategy to identify young children with attention-deficit/hyperactivity disorder [5]. The reason for this recommendation was twofold: First, the early debut of ADHD symptoms is associated with negative consequences for the children and their families. Second, preschool intervention programs show promise in ameliorating symptoms [6, 7]. However, to date, a few pre-schoolers with ADHD symptoms have been offered interventions. One reason may be that hyperactive–impulsive symptoms are common in early childhood and have been found to decrease between the ages of 3 and 8 years in the general population [8], and inattentive symptoms may be difficult to detect [9]. Thus, early identification of ADHD through screening programs carries the inherent problem of misclassification, making it difficult to accurately separate children with persistent high levels of ADHD symptoms from those who will outgrow their problems. There is a need to investigate the screening of persistent high ADHD levels from preschool to school age.

A major challenge in ADHD screening at preschool age, with reasonable sensitivity (often set to ≥ 70% [10]), is the risk of also identifying many false-positive children. This would cause unnecessary concern for many families and increase the strain on healthcare services. Thus, repeated screening and multiple thresholds have been suggested [10, 11], but they need empirical support. A recent Chinese study of pupils aged 6–12 years found that a two-stage screening involving teachers (adding teacher interviews to traditional questionnaire-based screening) increased specificity from 80 to 93% while maintaining sensitivity at 83%, thus reducing the proportion of false positives and improving the clinical utility of school-based screening for ADHD [12]. That study recommended a two-stage screening process but concluded that further research is required to identify the optimal approach to screening for ADHD.

The present study aims to test a parent-reported two-stage-screening for ADHD at ages 3.5 and 5 years to identify children with persistent high levels of ADHD symptoms from preschool to school age. We hypothesized that this approach would be more accurate than a single screening at 3.5 years of age.

## Method

### Participants

The Norwegian Mother, Father, and Child Cohort Study (MoBa) is a population-based cohort study conducted by the Norwegian Institute of Public Health. From 1999 to 2008, pregnant Norwegian-speaking women having their first ultrasound were enrolled from all over Norway (*n* ~ 114.500 children; 41% participation rate) [13]. Nested within the MoBa is the ADHD substudy, which oversampled for children at risk using 11 items about ADHD from the MoBa questionnaire when the child was 3 years old. This study has previously been described in detail [14, 15]. About 80% of the invited participants (*n* = 2798) had scores ≥ 90th percentile on these 11 items. The rest were randomly selected children (n = 654) from MoBa. Thirty-five percent agreed to participate, and from 2007 to 2011, 1195 children (mean age 3.5 years) took part in a 1-day clinical assessment, including a diagnostic interview with their parents (mainly mothers). Fifteen mothers later withdrew from MoBa, leaving 1180 enrolled children who were followed up at 5 and 8 years of age. This study includes children with available screening data at 3.5 and 5 years of age, and information to define the ADHD outcome groups at 3.5, 5, and 8 years (*n* = 707).

### Measures

Child sex was obtained from the Norwegian Medical Birth Registry.

### Screening at 3.5 and 5 years of age

At 3.5 and 5 years, we used six items from the Child Behavior Checklist (CBCL)/1.5–5 Diagnostic and Statistical Manual of Mental Disorders (DSM)-oriented scale for ADHD (Can’t concentrate, Can’t sit still; Can’t stand waiting; Demands must be met immediately; Gets into everything; Quickly shifts activities) [16]. Mothers rated the CBCL items on a three-point Likert scale (not true, somewhat true, or very true; range 0–2).

A total of 707 children, 381 boys and 326 girls, had complete data. A bias test for attrition at 3.5, 5, and 8 years did not show any significant differences between included and lost children with respect to sex or percentage above ADHD threshold at any point in time. At 3.5 years of age, those lost to follow-up had a slightly higher mean CBCL score (5.49 vs 5.11; *p* < 0.05); while there was no significant difference at 5 years of age.

### ADHD outcome at three time points (See Fig. 1)

**Fig. 1:**
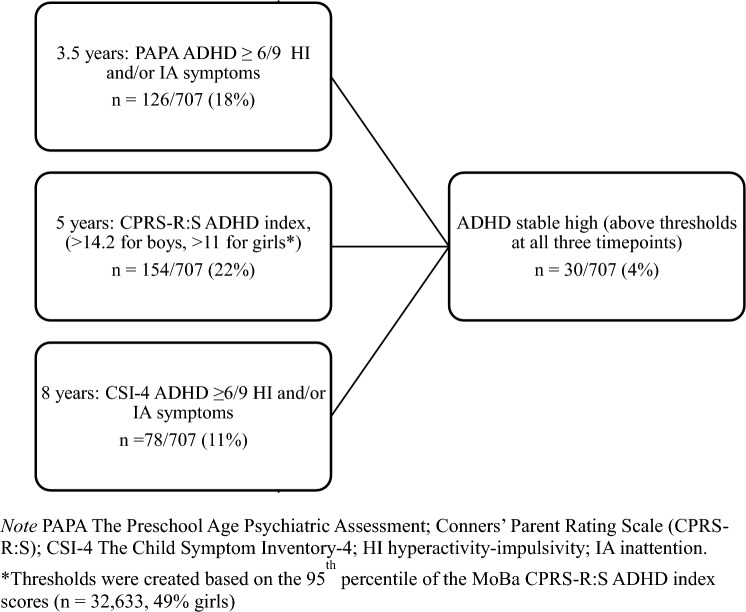
Illustration of the ADHD measures and thresholds used at the different timepoints

At 3.5 years of age, the semi-structured Preschool Age Psychiatric Assessment (PAPA) interview [17] was developed for children from the ages of 2 to 5 years. The interviewer asks questions until they can decide whether the symptoms described meet the definitions provided in a glossary. A PAPA reliability study reported a test–retest intraclass correlation of 0.80 for classified ADHD [18]. In the present study, only ADHD symptoms persisting for ≥ 3 months were counted as present. In line with our earlier studies [14, 15], we used information from the PAPA, and defined ADHD by the DSM-IV-TR criteria, with at least six out of nine symptoms of hyperactivity–impulsivity and/or inattention [19]. A second blind rater rescored audiotapes of 79 randomly selected interviews, and the intraclass correlation was 0.98 for ADHD symptoms. Nineteen percent (222/1180) of the children met the symptom criteria for ADHD.

At 5 years of age, the mothers received the revised short form of the Conners’ Parent Rating Scale (CPRS-R: S) with 12 ADHD items comprising the ADHD index [20], as part of the 5-year MoBa questionnaire. The CPRS items were rated on a four-point Likert scale (not true, somewhat true, often true, or very true; range 0–3). The ADHD index has been found to have good validity for ADHD [21]. From all the responders to the 5-year MoBa questionnaire (*n* = 32,633, 49% girls), we created thresholds for ADHD to be present at the 95th percentile of the CPRS-R:S ADHD index scores: > 14.2 for boys and > 11 for girls. Using thresholds from the general population from which the children were drawn, reduced the problem of cross-cultural differences if using US cut-of scores, and is in line with a previous study [22]. Of the 1180 children, 966 (82%) participated at the age of 5 years, and 220 of these (23%) reached the ADHD classification based on these thresholds.

At 8 years of age, parents responded to the Child Symptom Inventory-4 (CSI-4), rated on a four-point Likert scale (never, sometimes, often, very often; range 0–3) [23]. We used the hyperactivity–impulsivity and inattention subscales, each with nine items, and dichotomized symptom counts where symptoms were scored as not present (never/sometimes = 0) or present (often/very often = 1). In line with the CSI-4 manual, children who reached the minimum number of symptoms necessary for the DSM-IV ADHD diagnosis with ≥ 6 on either subscale were classified with ADHD. Sixty-six percent (783/1180) of the participants at 3 years of age had data on the CSI-4 at 8 years of age. Of these children, 85 (11%) were classified with ADHD.

### Ethics

MoBa and the initial data collection were based on a license from the Norwegian Data Protection Agency and approval from the Regional Committees for Medical and Health Research Ethics. The MoBa cohort is currently regulated by the Norwegian Health Registry Act. The current study was approved by the Regional Committees for Medical and Health Research Ethics (2017/1276).

### Analytic plan

We divided the children into four groups based on the number of times (0, 1, 2, or 3) they had been classified with ADHD. We compared the mean ADHD scores for the groups with an analysis of variance with pairwise post hoc comparisons using Scheffe’s test. Because there were different ADHD outcome measures at ages 3.5, 5, and 8 years, we used *z*-transformation to obtain comparable scores. We then tested the ability of the CBCL DSM-oriented scale for ADHD to discriminate the ADHD persistent group (above threshold at all three time points) from the rest. With receiver-operating characteristic (ROC) analyses, we estimated the areas under the curves (AUCs) to quantify the overall accuracies of the single- and two-stage screening. The ROC curve graphically represents the probability of true positive results of ADHD as a function of the probability of false-positive results. For interpreting AUC values, the following guideline is recommended: < 0.70 = poor, 0.70–0.79 = fair, 0.80–0.89 = good, and 0.90–1.00 = excellent [24]. We estimated the CBCL scales’ sensitivity (Se; the probability that a measure correctly classifies a case as positive) and specificity (Sp; the probability that a measure correctly identifies non-cases as negative) for thresholds 6, 7, 8, and 9. Children who scored at or above a given threshold score were categorized as screen positive. We calculated the positive predictive values (PPVs, the probability of a true case given a positive test), negative predictive values (NPVs, the probability of a true non-case given a negative test), the positive-likelihood ratios (LRs, the probability of a child who has the disorder testing positive divided by the probability of a child who does not have the disorder testing positive), and negative LRs (the probability of a child who has the disorder testing negative divided by the probability of a child who does not have the disorder testing negative). LRs greater than 1 suggest the presence of the disorder being present, whereas LRs between 0 and 1 indicate its absence. LRs equal to 1 lack diagnostic value [25]. LRs are derived from the Se and Sp values and are independent of the proportion of the disorder in the sample, thereby increasing the likelihood of generalizability to other samples [26]. We checked whether sex altered the proportion with ADHD correctly classified.

## Results

**Fig. 2:**
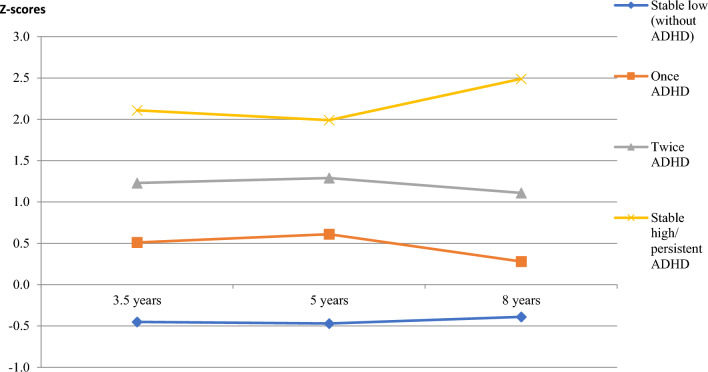
ADHD Z-scores at 3.5, 5, and 8 years of age for the different ADHD groups

Of the 707 children, 126 (18%) were classified with ADHD at 3.5 years of age, 154 (22%) at 5 years, and 78 (11%) at 8 years. Thirty (4%; 19 males) were classified with ADHD at all three time points, 74 (11%) twice, and 120 (17%) only once. Pairwise comparisons showed significant differences between ADHD scores of the groups shown in Fig. 2 (*p* < 0.001 for all).

**Fig. 3:**
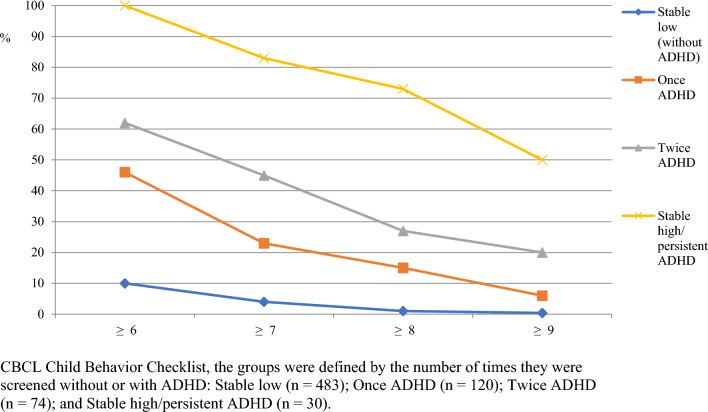
Percentages of screen positives by two-stage screening (3.5 and 5 years of age) for different CBCL thresholds by the ADHD groups

Figure 3 shows the percentages of screen positives for the ADHD groups by two-stage CBCL screening at different thresholds.

For both the single- and two-stage screening, the CBCL discriminated the children classified with persistent ADHD from the negative cases significantly better than chance (*p* < 0.001), both performing excellently (AUCs = 0.90 and 0.94, respectively).

Two-stage screening with a threshold of 6 identified all 30 children within the persistent ADHD group, but also 150 children within the other groups (specifically, 62%, 46%, and 11% of the children classified with ADHD twice, once, and never (Fig. 2)). Increasing the screening threshold to 9 identified half of the children within the persistent ADHD group (*n* = 15), but also reduced false positives considerably (*n* = 24), improving PPV (from 0.17 to 0.38) (Table 1).

**Table 1:** Prediction values for different CBCL thresholds when administrating single (at 3.5 years of age) or two-stage screening (at 3.5 and 5 years of age) to identify the persistent ADHD group

CBCL threshold scores	TP	FP	FN	TN	Se (%)	Sp (%)	PPV	NPV	+ LR (CI)	–LR(CI)
Single screening										
6	30	263	0	414	100	61	0.10	1.00	2.57 (2.34–2.83)	0.00
7	27	160	3	517	90	76	0.14	0.99	3.81 (3.18–4.56)	0.13 (0.04–0.38)
8	25	105	5	572	83	84	0.19	0.99	5.37 (4.24–6.82)	0.20 (0.09–0.44)
9	21	65	9	612	70	90	0.24	0.99	7.29 (5.25–10.13)	0.33 (0.19–0.57)
Two-stage screening										
6	30	150	0	527	100	78	0.17	1.00	4.51 (3.92–5.20)	0.00
7	25	78	5	599	83	88	0.24	0.99	7.23 (5.56–9.41)	0.19 (0.08–0.42)
8	22	41	8	636	73	94	0.35	0.99	12.11 (8.39–17.48)	0.28 (0.16–0.51)
9	15	24	15	653	50	96	0.38	0.98	14.10 (8.29–24.00)	0.52 (0.36–0.74)

*CBCL* child behavior checklist; *TP* True positives; *FP* false positives; *FN* False negatives; *TN* true negatives; *Se* sensitivity; *Sp* specificity; *PPV* positive predictive value; *NPV* negative predictive value; + LR positive-likelihood ratio; –LR negative-likelihood ratio; *CI* confidence interval

Table 1 shows the discrimination for each CBCL threshold for single- and two-stage screenings. At a threshold of 6, both single- and two-stage screenings gave perfect sensitivity (100%). However, the two-stage screening lowered the number of false positives by 113 children, and thus increased specificity. When requiring sensitivity values of at least 70%, the single screening with a threshold of 9 gave a high probability of correctly identifying children within the persistent ADHD group (+ LR = 7.29) but missed 9 of the 30 children and identification 65 false positives (PPV = 0.24). Two-stage screening with a threshold of 8 seemed to give the best overall trade-off, with an acceptable sensitivity of 73% (Sp = 94%), loss of eight true positives, and identifying of 41 false positives (PPV = 0.35). In comparison, the PPV at a threshold of 8 from a single screening (3.5 years of age) was 0.19. At this threshold, we also checked the single screen values at 5 years and found similar values to those at 3.5 years (PPV = 0.19). There were only marginal and nonsignificant sex differences in screening accuracies (statistics not shown).

## Discussion

In the present study, we showed that even though a considerable proportion of the children were classified with ADHD at least once across time, only a small minority were classified with ADHD at all three time points (ages 3.5, 5, and 8 years), comprising the persistent ADHD group. For population-based studies, having persistent ADHD as an outcome should be a requirement when evaluating screening accuracies in young children because ADHD-like behavior is normal in this developmental period. With persistent ADHD as the outcome, we showed that both screening at 3.5 years of age (single) and at 3.5 and 5 years (two-stage screening) with the CBCL had excellent overall accuracies. However, the two-stage screening was more accurate than single screening at all thresholds, mainly due to a substantial reduction in false-positive rates. This knowledge may be useful to limit unnecessary concern for families and to avoid placing strain on healthcare services.

Nearly 20 years ago, a review underlined that defining the boundaries between normal and clinically significant hyperactivity–impulsivity and inattention is challenging in pre-schoolers as inhibiting behavior and sustaining attention is under development [27]. This may explain the finding that only a small proportion of children in the study sample were persistently classified with ADHD at 3.5, 5, and 8 years of age. Previously, instability of ADHD symptoms from preschool to school age has been found most pronounced in population studies [8, 9, 28–30] but also in clinical studies [31–33]. One German study reported low-to-moderate ADHD stability during 1 year in kindergarten [30]. Similarly, we previously reported that only 47% (45/97) of the 3 years old classified with ADHD by a diagnostic interview, reached the threshold for ADHD at 8 years of age [9]. Together, these studies support the need to focus on persistent ADHD when evaluating screening tools during preschool. Previous cross-sectional preschool studies have been promising in reporting good psychometric properties of different ADHD screening measures [34–36], including acceptable screening accuracies [14, 15, 37–39]. The present study used the well-validated and clinically much used CBCL DSM-oriented scale for ADHD [16] in line with one study (*n* = 616) showing good-to-excellent discriminative capacities of the CBCL to identify ADHD measured with a diagnostic interview between 3 and 5 years of age [40]. However, none of the above-mentioned studies followed the children over time. In addition, a recent meta-analysis of ADHD screening tools concluded that although most have excellent overall diagnostic accuracy, a single measure is unlikely to have sufficient sensitivity and specificity for clinical use or population screening [11]. Assuming that the correct ADHD prevalence rate during preschool years lies between 1.9 and 5.7% [27, 41], these tools will identify an overwhelming number of false-positive children, which could cause strain to many families and be costly to society.

We found that two-stage screening reduced the false-positive rates considerably at all thresholds. This finding was in line with the only previous study using two-stage screening involving teachers to identify ADHD in school children [12]. In clinical practice, it is essential to reach as many at-risk pre-schoolers as possible (highest possible sensitivity) to ameliorate symptoms and improve outcomes, but without unduly raising concern. In the present study, a threshold of 6 in both the single- and two-stage CBCL screening identified all children in the persistent ADHD group (100% Se). However, the two approaches differed in the crude number of false positives and thus in specificity (61% and 78%, respectively). To illustrate, the single screening with a threshold of 6 identified 239 (263—24) more false-positive pre-schoolers than the two-stage screening with a threshold of 9. For most clinics, unnecessary follow-up of many healthy children (with extensive assessments and perhaps treatment) is unacceptable. Repeating the screening at 5 years of age, and raising the thresholds, appears to be the way forward to reduce false positives. This is in line with a review on screening, which pointed out that it would not be costly to refer all children if thresholds are set extremely high, because very few would be identified [10]. However, in the present study, we showed that a single screening with the highest threshold would not be strict enough to avoid several false positives (*n* = 65), perhaps bringing psychological harm without reason [42]. Depending on resources and on how children are followed up (e.g., interview with parents before school entry), the optimal screening approach in our study was the two-stage screening with a threshold of 8, with the loss of eight at-risk children, and only 41 false positives.

### Strengths and limitations

The strengths of the present population-based cohort study were the large sample, the longitudinal design, and the clear definition of a group of children with persistent ADHD with valid and reliable measures [18, 20, 43]. There were also several limitations. Although there were selection biases due to attrition [13], one MoBa-study reporting on ADHD found small differences and assumed limited effects on generalizability [44]. The sampling procedure at 3 years of age, increased the ADHD occurrence rates compared with the general population, possibly inflating the predictive values. There were few children in the persistent group, raising the concern that we may have excluded children with ADHD onset after the age of 3 years. However, our screening procedure at ages 3.5 and 5 years was not designed for detecting such a group with later onset, which would also have required a longer follow-up time to be more certain of stability. Different measures for ADHD at different time points made it impossible to directly compare scores across time, but by calculating z-scores, we were able to follow the children’s deviations from the mean across time points. We only had parent information available in the present study. While a recent review recommends the use of parent-reported measures for screening in young children [11], because we only had clinician assessments of the parent information at 3.5 years of age (PAPA), and otherwise relied on parent questionnaires (from mothers), we may have overestimated the screening accuracies compared with using the outcome of clinically diagnosed ADHD.

## Conclusions and future directions

To conclude, this study shows that preschool two-stage screening is more accurate than single screening in identifying children classified with persistent ADHD, mainly by substantially reducing the number of false-positive children. Clinicians who identify pre-schoolers with high symptom scores on ADHD screeners should repeat the screening after some time to see if the symptoms persist before referring children to time-consuming and costly assessments. Future studies should use persistent ADHD as the outcome when estimating the accuracies of ADHD screening tools in young children and should investigate the cost–benefit of such screening. Two-stage screening is recommended to identify pre-schoolers most accurately with ADHD, mainly due to the reduction in the number of false-positive children. This approach may reduce concern for many families and the strain on to healthcare services.

## Data Availability

The data that support the findings of this study were available from MoBa at the Norwegian Institute of Public Health, but restrictions apply to the availability of these data, used under license for the current study, and so are not publicly available.
